# mm-Wave Radar-Based Vital Signs Monitoring and Arrhythmia Detection Using Machine Learning

**DOI:** 10.3390/s22093106

**Published:** 2022-04-19

**Authors:** Srikrishna Iyer, Leo Zhao, Manoj Prabhakar Mohan, Joe Jimeno, Mohammed Yakoob Siyal, Arokiaswami Alphones, Muhammad Faeyz Karim

**Affiliations:** 1 School of Electrical and Electronic Engineering, Nanyang Technological University, Singapore 639798, Singapore; srikrish001@e.ntu.edu.sg (S.I.); manoj.mohan@ntu.edu.sg (M.P.M.); eyakoob@ntu.edu.sg (M.Y.S.); ealphones@ntu.edu.sg (A.A.); 2 SCALE @ NTU Corp Lab, Nanyang Technological University, Singapore 639798, Singapore; leo.zhao@ncs.com.sg (L.Z.); joe.jimeno@ncs.com.sg (J.J.); 3 NCS Group, Singapore 469272, Singapore

**Keywords:** mm-wave radar, artificial neural network, vital signs, machine learning

## Abstract

A non-contact, non-invasive monitoring system to measure and estimate the heart and breathing rate of humans using a frequency-modulated continuous wave (FMCW) mm-wave radar at 77 GHz is presented. A novel diagnostic system is proposed which extracts heartbeat phase signals from the FMCW radar (reconstructed using Fourier series analysis) to test a three-layer artificial neural network model to predict the presence of arrhythmia in individuals. The effect of person orientation, distance of measurement and movement was analyzed with respect to a reference device based on statistical measures that include number of outliers, mean, mean squared error (MSE), mean absolute error (MAE), median absolute error (medAE), skewness, standard deviation (SD) and R-squared values. The individual oriented in front of the radar outperformed almost all other orientations for most distances with an expected d = 90 cm and d = 120 cm. Furthermore, it was found that the heart rate that was measured while walking and the breathing rate which was measured for a motionless individual generated results with the lowest SD and MSE. An artificial neural network (ANN) was trained using the MIT-BIH database with a training accuracy of 93.9 % and an R^2^ value = 0.876. The diagnostic tool was tested on 15 subjects and achieved a mean test accuracy of 75%.

## 1. Introduction

Developing continuous vital sign monitoring systems has been identified by healthcare institutes as necessary to safeguard the health of seriously ill patients or infants in hospitals or at home [1,2,3,4]. Current systems of monitoring heart rate variability (HRV) involve the usage of electrocardiography (ECG) electrodes, pulse oximeter, photoplethysmography (PPG) and wearable devices such as the OMRON^TM^ 10 series. Breathing rate is measured manually using a timer or using oronasal sensors, which measure fluctuations in air pressure due to respiration [5]. These traditional methods can be inaccurate due to random body movements (RBM) and can cause discomfort for users, especially when used for ambulatory monitoring. Recently, radar-based solutions have been proposed for non-contact measurement of heart rate and respiration rate. The most popularly used radars include continuous-wave (CW) Doppler radars [6,7,8,9,10,11], impulse radio ultra-wideband (IR UWB) radars [12,13] and frequency-modulated continuous wave (FMCW) Doppler radars [14,15,16,17]. Lazaro et al. [18] presented a feasibility study on an IR-IWB radar-based vital sign monitoring system. However, the weak heart signal was difficult to isolate from external noise, respiratory harmonics and third-order intermodulation products, which can lead to inaccurate heart rate readings. In [19], a novel radar hardware system is proposed that utilizes a sweeping correlation method, which applies a small-frequency difference to the received impulse train, thereby increasing repetition frequency and measurement accuracy. However, the proposed method has only been used to detect respiratory waves. Additionally, IR UWB radars have long been used in several wireless sensing use cases, such as people counting and detection [20], human detection through walls [21], home monitoring and remote care systems [22] and classification of humans and animals based on vital signs [23]. Since the power transmitted by the pulse (the IR-UWB radars transmit multiple short Gaussian pulses without a carrier frequency) increases the signal-to-noise ratio, it also necessitates a high-frequency analogue-to-digital converter (ADC), which increases the cost, power consumption and complexity of design [24]. CW radars are only capable of detecting relative displacements using phase differences; hence, they are incapable of localizing objects when multiple moving objects are around. However, FMCW radars are capable of localizing objects based on changes in frequency and phases between transmitted and received chirps [25,26].

FCMW radars overcome the shortcomings of CW radars, as extremely high frequency (EHF) radar systems operating in the 110–300 GHz frequency band can detect displacements in the order of cm-mm and become absorbed on the skin of an individual without causing any harmful biological effects [27,28]. Moreover, the MIMO antenna framework allows us to localize multiple targets; hence, we are able to monitor multiple moving targets simultaneously using beamforming techniques [17]. In [29], a TI-AWR1443 FMCW radar operating in the 77 GHz band is utilized to detect vital signs for a person lying on a bed using the phase-unwrapping algorithm proposed in [30]. It was found that the regression coefficient values of BR and HR values were 0.883 and 0.64 when compared to the reference hexoskin contact sensor [31].

In most studies, radars are solely used for monitoring heart rate and breathing rate. In this work, we used a three-layered artificial neural network to predict the onset of arrhythmia based on statistical features extracted from the phase signals of a localized range bin using an FMCW radar. Additionally, a signal reconstruction module is proposed that helps generate phase signals with higher signal-to-noise ratio (SNR) values. The radar was also tested in comparison to a reference device, i.e., OMRON, to analyse and compare the effect on vital signs’ detection due to varying person orientation, distance of measurement and random body movements.

This paper is organized as follows. In Section 2, the mathematical background, algorithmic workflow of the system and the proposed prediction flow for arrhythmia are described. The results of the experiments are discussed in detail in Section 3. Finally, the advantages and potential future work is outlined in Section 4.

## 2. Materials and Methods

**Figure 1:**
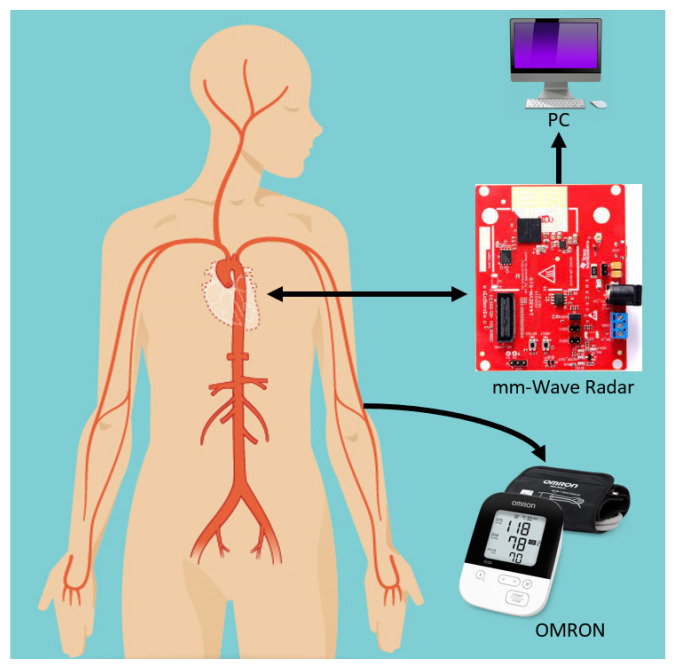
Schematic of the proposed mm-wave radar system: an mm-wave radar is fixed at front of the subject while an Omron sphygmomanometer is attached, which simultaneously extracts pulse readings for verification. The radar data are recorded on a PC through a USB connection.

The system setup consisted of Texas Instrument (TI) IWR1443BOOST FMCW radar-based evaluation module, Omron device connected to a personal computer (PC) as shown in Figure 1. TI IWR IWR1443BOOST operates in the 76–81 GHz band with 3 transmitting antennas and 4 receiving antennas.

### 2.1. FMCW Radars 

**Figure 2:**
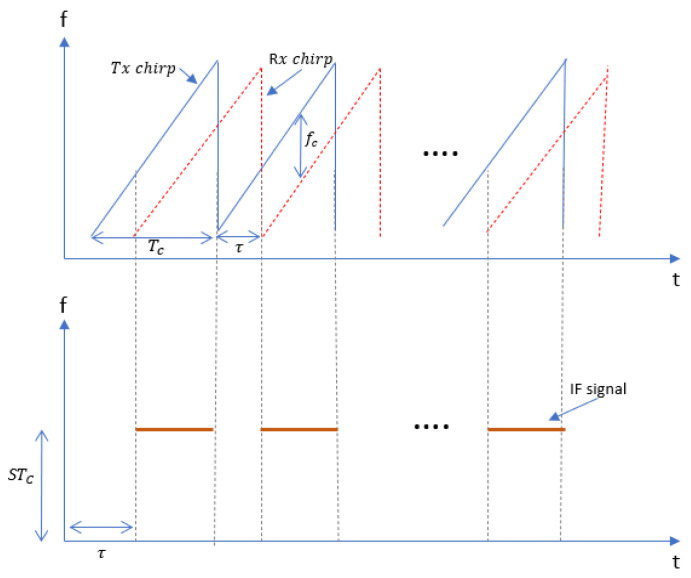
Transmitted and received chirp signals where each chirp is a sinusoidal signal with a slope S and time delay between transmitted and received chirp τ when the sweeping bandwidth is *S*τ.

Frequency-modulated signals are robust against additive noise such as thermal noise unlike amplitude-modulated waves. In [17], FMCW transmitted and received chirps were modelled as increasing ramp functions as shown in Figure 2. The transmitted signal is given by:

(1) $$x_{T}\left(t\right)=A_{T}\cos\left(2\pi f_{c}t+\pi \frac{B}{T_{C}}t^{2}+\theta \left(t\right)\right)$$

where $A_{T},\;f_{c},B,T_{C}$ are the amplitude of the transmitted signal, chirp start frequency and bandwidth of chirp and chirp duration, respectively.

Since we are measuring vital signs of a single target within the field of view, we assume a single reflection model where the received chirp given by Equation (2) is scaled by factor β and time shifted by τ.

(2) $$x_{R}\left(t\right)=\beta A_{T}\cos\left(2\pi f_{c}\left(t-\tau \right)+\pi \frac{B}{T_{C}}{\left(t-\tau \right)}^{2}+\theta \left(t-\tau \right)\right)$$

Here, τ=2R(t)/c is the time delay from the subject. R(t) is the time-dependent radar range.

**Figure 3:**
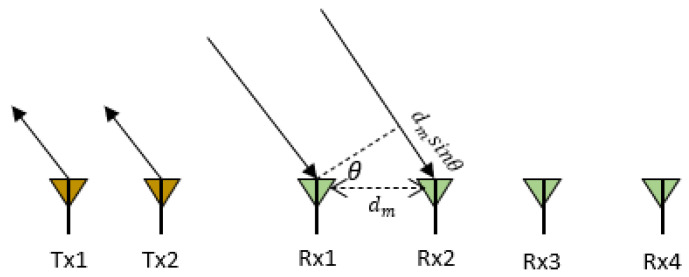
A MIMO radar with two transmitting antennas (Tx) and four receiving antennas (Rx) where distance between two receiving antennas is $d_{m}$ and θ is the angle of arrival.

The intermediate frequency signal after I/Q mixing is approximated as

(3) $$\begin{array}{c} y\left(t\right)=P_{R}e^{j(2\pi \left[\frac{B}{T_{C}}\tau \right]t+2\pi f_{c}\tau +\pi \frac{B}{T_{C}}\tau^{2}+\Delta \theta \left(t\right)}\\ \;\;\approx \;P_{R}e^{j\left(2\pi f_{b}t+2\pi f_{c}\tau \right)} \end{array}$$

where beat frequency $f_{b}=\frac{B}{T_{C}}\tau$, Δθ(t) is residual phase noise, which can be neglected for our short range (<1.5m) detection experiments due to the range correlation effect [32]. Additionally, the additional term $\pi \frac{B}{T_{C}}\tau^{2}$ is negligible, so it can be neglected. Hence, the IF signal for the kth ADC sample and lth chirp is given by:

(4) $$y\left(k,l\right)=P_{R}e^{j\left(2\pi f_{b}kT_{f}+\frac{4\pi }{\lambda }R\left(kT_{f}+lT_{s}\right)\right)}$$

where $P_{R}$, $f_{b},\;T_{f}$, $T_{s}$ are the received signal power, beat frequency, sampling time of fast-time axis and sampling time of slow-time axis, respectively. To improve the angular resolution, a time division multiplex multiple-input multiple-output (TDM-MIMO) radar system is used that consists of 2 transmitting and 4 receiving antennas. Since $d_{m}\ll R\left(t\right)$ and assuming a planar wavefront, the received wave must travel an additional distance of $d_{m}\sin\Theta$ as shown in Figure 3.

Thus, an additional phase shift between the receivers and the beat signal is given by:

(5) $$y\left(k,l\right)=P_{R}e^{j\left(2\pi f_{b}kT_{f}+\frac{4\pi }{\lambda }R\left(kT_{f}+lT_{s}\right)+\frac{2\pi d_{m}\sin\Theta }{\lambda }\right)}$$

where the receiving antennas are $d_{m}$ distance apart. Hence, the phase shift at mth receiver is given by:

(6) $$\Phi_{m}=\frac{4\pi R\left(k\;T_{f}+lT_{s}\right)}{\lambda }+\frac{2\pi d_{m}\sin\Theta }{\lambda }$$

As we measure displacements of < 5 mm, frequency < 2 Hz and a single target in the same range bin, there would be no change in phase across the fast-time axis, i.e., phase change will be constant. Therefore, $R\left(k\;T_{f}+lT_{s}\right)\;\approx R\left(k\;T_{f}\right)+R\left(lT_{s}\right)$ gives us:

(7) $$\Phi_{m}=\frac{4\pi R\left(k\;T_{f}\right)}{\lambda }+\frac{4\pi R\left(lT_{s}\right)}{\lambda }+\frac{2\pi d_{m}\sin\Theta }{\lambda }$$

(8) $$\Phi_{m}=\Phi_{T_{f}}+\frac{4\pi R\left(lT_{s}\right)}{\lambda }+\frac{2\pi d_{m}\sin\Theta }{\lambda }$$

where $\Phi_{T_{f}}$ is constant. We are mainly concerned with phase changes along the slow-time axis. However, changes in $\Phi_{T_{f}}$ can adversely affect our results so all our experiments (except Section 3.3) were carried out for stationary subjects only.

### 2.2. Process Flow for the Detection of Vital Signs

**Figure 4:**
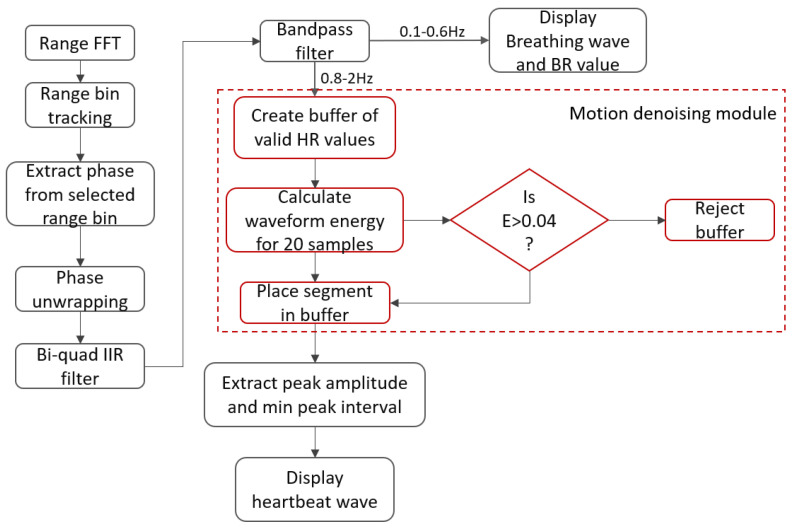
Signal processing workflow: after applying the range FFT, for a single range bin, the angle FFTs are computed, upon which the phase shift is used to extract the heartbeat and breathing waves.

The process flow for the detection of the vital signs is shown in Figure 4. As described in [17], each chirp signal sampled at the beat frequency $f_{b}$ is converted to a complex range profile by applying the range fast Fourier transform (FFT). Range profiles of multiple chirp signals are stacked on top of each other and converted into a matrix with *i* number of rows (fast time samples) and *j* columns (slow-time samples). As summarized in Table 1, the slow-time axis rate is 20 chirps/sec where the duration of the chirp is 50 µs. Since vital signs are detected for a stationary person, the phase change across the slow-time axis is extracted from a single range bin. The phase-unwrapping algorithm is then implemented to unwrap the phase beyond (−π,π).

Next, the phase values are filtered using a serially cascaded Bi-Quad Infinite impulse response (IIR) filter into the cardiac frequency spectrum of (0.8–2) Hz and the breathing frequency spectrum of (0.1–0.5) Hz. The motion denoising module computes the energy of the waveform for a window size of 1 sec and discards the windowed waveform if it exceeds a threshold of E_th_ = 0.04. The maximum and minimum peak-to-peak distance threshold is automatically computed based on the mean of all distances. A peak is rejected if it is out of 1 standard deviation from the mean. Finally, the breathing rate and heart rate are computed based on the frequency of filtered peaks within their respective frequency spectrums as mentioned earlier. Additionally, for our experiment, we propose a QRS complex generation module, which extracts the signal peaks and base period of the heartbeat phase signal to mimic QRS-based radar heartbeat signals using Fourier series representation of triangular waveforms, thus eliminating signal overshoots, aperiodicity and improving the SNR value of the extracted signal as described in the next section. The QRS complex refers to a combination of Q, R and S waves, which represent the ventricular depolarization of the heart. The QRS complex is the most vital part of an ECG signal because it contracts the ventricles as the oxygenated blood from the left ventricle is pumped out from the heart to other parts of the body, which corresponds to maximum electrical activity (highest voltage amplitude). Our proposed system detects R peaks that have the largest amplitude within a QRS complex to extract statistical features based on the peak-to-peak interval (RR interval) of the ECG signal sequence.

### 2.3. Heartbeat Signal Generation

**Figure 5:**
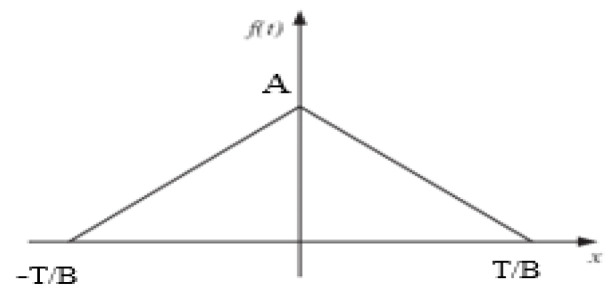
Symmetric triangular wave function to model the QRS components of an ECG signal.

An ECG signal is a periodic wave signal that satisfies the Dirichlet conditions. It can be modelled as a combination of scaled amplitude and multiples of fundamental frequency of sinusoidal and cosine functions using Fourier series expansion. The QRS components can be modelled by a symmetric triangular wave function as shown in Figure 5. Consider the even symmetric triangular wave function given by (9):

(9) $$f\left(t\right)=\{\begin{array}{c} -\frac{BAt}{T}+A,\;0<t<\frac{T}{B}\\ \frac{BAt}{T}+A,-\frac{T}{B}<t<0 \end{array}$$

where *T* is the time period, *A* is the amplitude, and *B* is the factor which determines the QRS interval.

Since f(t) is an even function, $b_{n}=0$. Fourier series coefficients are formulated as:

(10) $$a_{o}=\frac{1}{T}\int_{-T}^{T}f\left(t\right)dt=\frac{2}{\frac{T}{B}}\int_{0}^{\frac{T}{B}}-\frac{BAt}{T}+A\;dt=\frac{A}{B}\left(2-B\right)$$

(11) $$a_{n}=\frac{2B}{T}\int_{0}^{\frac{T}{B}}f\left(t\right)\cos\left(nwt\right)dt=\frac{2B}{T}\int_{0}^{\frac{T}{B}}-\frac{BAt}{T}+A\cdot \cos\left(nwt\right)dt=\frac{2BA}{n^{2}\pi^{2}}\cdot \left(1-\cos\left(\frac{n\pi }{B}\right)\right)$$

Hence, Fourier series coefficients can be substituted in (12).

(12) $$f\left(x\right)=\frac{a_{0}}{2}+\overset{\infty }{\underset{n=1}{\sum }}a_{n}\cos\left(\frac{n\pi x}{T}\right)$$

where n=1,2,3,… which is the multiple of fundamental frequency.

### 2.4. Arrhythmia Detection Using Neural Networks

This work proposes a cardiac disorder diagnostic scheme using a 3-layer neural network model. The model is trained using ECG signals which lie in the frequency range 0.05~100 Hz, and its maximum amplitude is 5 mV. ECG signals are extracted using electrodes mounted on the body. Hence, some artifacts are filtered out before statistical features can be extracted from the ECG signal database. Artifacts include but are not limited to muscle tremor, electromagnetic interference (EMI) and base-line wander. Muscle tremor artifacts caused due to shivering or sudden body movements (usually in the elderly) are high-frequency signals at 30~300 Hz that are removed by Butterworth low-pass filters. The 50 Hz electromagnetic interference is suppressed by a Butterworth band-stop filter. Lastly, baseline wander is an ultra-low frequency signal that ranges between 0 and 0.8 Hz that can be eliminated using a high-pass filter.

**Figure 6:**
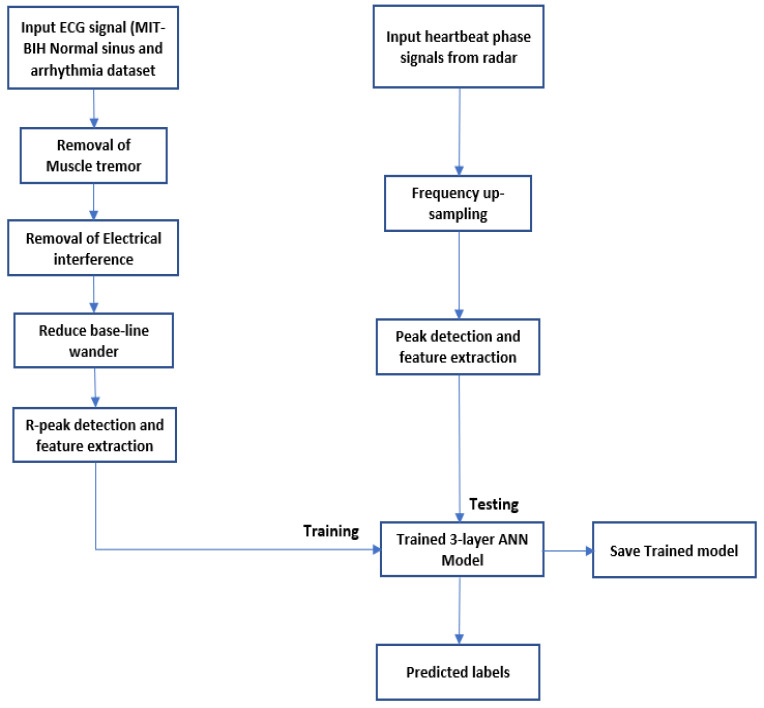
Predict the onset of arrhythmia based on statistical features extracted from the phase signals of a localized range bin using an FMCW radar.

**Figure 7:**
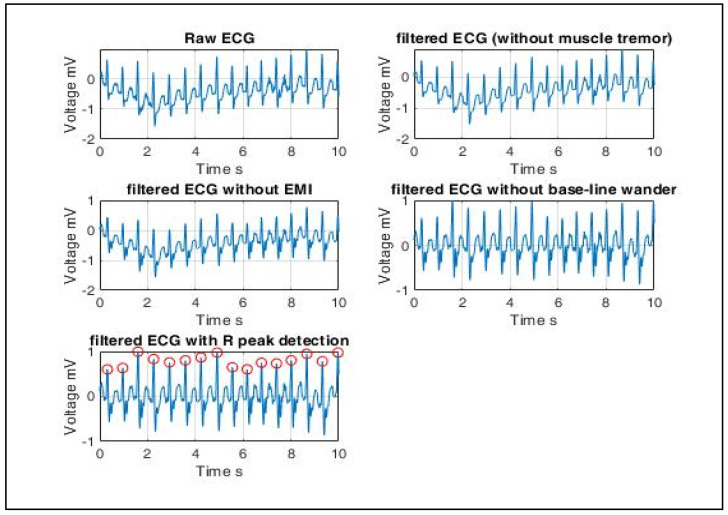
ECG signal pre-processing (left to right): ECG signals are extracted using electrodes mounted on the body. Hence, some artifacts are filtered out before statistical features can be extracted from the ECG signal database. Artifacts include muscle tremor, electromagnetic interference (EMI) and base-line wander. Muscle tremor artifacts caused due to sudden body movements are high-frequency signals (30~300 Hz) that are removed by Butterworth low-pass filters. The 50 Hz electromagnetic interference is suppressed by a Butterworth band-stop filter. Baseline wander is an ultra-low frequency signal that ranges between 0 and 0.8 Hz that can be eliminated using a high-pass filter. Finally, the resultant R peaks of a QRS complex are detected, and only RR interval-based features are extracted since the radar-generated heartbeat phase signals are QRS equivalent signals.

As outlined in Figure 6, after filtering out low- and high-frequency noise, R peaks are detected as shown in Figure 7. The non-rhythmic ECG can be detected by extracting RR-interval-based features that include:

(13) $$\mathrm{Average}\;\mathrm{RR}\;\mathrm{interval}\;\mu_{RR}=\frac{\sum_{i=1}^{n}RR_{i}}{n}$$

(14) $$\mathrm{Normalized}\;\mathrm{maximum}\;\mathrm{difference}=\frac{\max\left(RR\right)-\min\left(RR\right)}{\mu_{RR}}$$

(15) $$\mathrm{Root}\;\mathrm{mean}\;\mathrm{square}\;\mathrm{of}\;\mathrm{successive}\;\mathrm{difference}\;RMSSD=\sqrt{\frac{\sum_{i-2}^{n}{\left(RR_{i}-RR_{i-1}\right)}^{2}}{n-2}}$$

(16) $$\mathrm{Coefficient}\;\mathrm{of}\;\mathrm{variation}=\frac{\sigma_{RR}}{\mu_{RR}}\;$$

(17) $$\mathrm{Normalized}\;\mathrm{absolute}\;\mathrm{deviation}=\frac{\sum_{i=1}^{n}\frac{RR_{i}-\mu_{RR}}{\mu_{RR}}}{n}$$

In addition to the above features, age and gender are included as training features. The dataset is then trained using a 3-layer neural network as described in Table 2, which consists of 8 and 16 neurons in the input and hidden layer, respectively. Weights are randomly initialized from the normal distribution function, and sigmoidal activation function is used. The mean square error is the loss function employed, which is backpropagated using the Levenberg–Marquardt algorithm to train the weights.

After peak detection of heartbeat phase signals obtained from the radar, training features given by (13)–(17) are extracted to test the trained model. Since we do not have a readily available radar-based signal database, it is important to note that we have restricted the application of our experiments solely to healthy individuals. However, to test the model for positive cases of arrhythmia, unseen signals from the MIT-BIH Arrhythmia dataset were used.

## 3. Results and Discussion

Our experiments were carried out using TI’s IWR1443BOOST FMCW radar-based evaluation module (EVM), which operates in the 76–81 GHz band with a total bandwidth of 4 GHz. The range profile, chest displacement, heartbeat and respiratory phase differences in addition to the heart rate (HR) and breathing rate (BR) values were displayed on a graphical user interface (GUI) designed using MATLAB R2020a. The radar was placed some distance apart in front of an individual, directly pointed towards their chest. Each observation lasting 25.6 s consisted of 128 data samples. Before we tested the radar for arrhythmia detection, the effect of orientation, distance from radar and movements were analysed and reported.

### 3.1. Measured Data Validation

Firstly, the heart rate values of the radar were validated based on their deviation from the HR values from our reference cuff-based OMRON device. The radar was placed 50 cm away from the individual and 20 observations were registered, i.e., for every 128 data samples generated by the radar, a corresponding OMRON HR value was registered. After every observation, 1 min of resting time was allotted before another observation was taken. The validation was evaluated based on the metrics described in Table 3. It was observed that the mean HR values were almost same for both the devices. The variance and standard deviation values of HR obtained by the OMRON device were lower than those of the radar. However, it is worth noting that the mean absolute error was only 3.85, i.e., HR values estimated by the radar only deviated by nearly ±4.

### 3.2. Effect of Orientation and Distance on Measurement

The radar was tested for different orientations, namely, front, back, right and left. For each orientation, the individual was seated in a stationary position at different distances away from the radar at 30, 60, 90, 120 and 150 cm. The 10 observations for HR and BR values were recorded for each orientation at varying distances. The number of outliers, mean, MSE, MAE, medAE, skewness and standard deviation were estimated to analyse the effect of orientation and distance. As shown in Table 4, boxes highlighted in orange and green are the best values obtained for HR and BR, respectively. The following observations were made:

- Range = 30 cm: As highlighted in green, the number of outliers for front and back were nil, while for right and left orientations the outliers had the greatest values. HR values (highlighted in yellow) when estimated in the front faired the best while analysis shows that BR was least skewed when obtained on the right side with minimized MSE and SD.
- Range = 60 cm: Again, the number of outliers for the front and back were nil while both the right and left suffered maximum skewness. The MSE and SD values of HR and BR were the least for the front, which performed the best.
- Range = 90 cm: Front and back orientations produced better results in terms of minimum outliers and lower SD values with an exception for the left position, which minimized skewness better.
- Range = 120 cm: Following the previous trend, right and left orientations produced poor results with the maximum number of outliers and highly skewed data. Again, individuals oriented in front of the radar outperformed other orientations.
- Range = 150 cm: We observed that some of the HR and BR values were estimated to be 0 as the radar was unable to pick up any chest displacements when the person was seated to the left or right. This is reflected in the analysis, which shows the presence of outliers and maximum skewness and SD.

To summarize, the results statistically prove that the left and right orientations are unsuitable for monitoring HR and BR. The radar performed well for the front and back orientation. However, the maximum range for monitoring an individual should be restricted to 120 cm as the problem of missing data arises beyond this range.

### 3.3. Effect of Movements on Measurement

The radar was placed at multiple distances from the individual, who was made to walk on a treadmill at 4.8 kmph (lowest speed). After one observation, a 1 min resting period was taken, after which the radar recorded the HR and BR values for the same individual while standing. The process was continued for nine more observations. As shown in Table 5, boxes highlighted in orange and green are the best values obtained for HR and BR, respectively. Based on the statistical analysis, the following observations were made:

- Range = 20 cm: The HR values while walking produced better results than standing as it generated no outliers and lesser standard deviation. While standing, the number of outliers, SD and MSE was lesser than that of values obtained when walking.
- Range = 40 cm: In this case, for both BR and HR, it is quite clear that best results were obtained when the person was standing.
- Range = 60 cm: Surprisingly, this range was observed to be the optimal distance for monitoring a moving person. The number of outliers was 0, and values were less skewed. Though BR values while walking had a higher deviation, it is important to note that the skewness value was almost negligible.

To summarize, we observed that the sensor could perform efficiently in generating low-skewed HR and BR values at a range of 60 cm when the person was moving. At range = 20 cm, results were inconclusive as one of the vital signs performed poorly for each activity. However, when patients moved to an optimum measuring distance of 40 cm, it became clear that individuals achieved better test results while standing without any movements as their MSE, MedAE, SD and skewness were lower.

#### 3.3.1. Validating Heart Rate Values during Movement

To make the performance analysis clearer, the HR values measured while standing and walking were compared to our reference OMRON device as described in Table 6. Where, the orange background indicates best HR values. After taking HR values for both scenarios, 1 min of resting time was given followed by measurement using OMRON. Hence, all measurements were taken with equal amounts of resting time.

As discussed before, HR values for the 40 cm range performed as expected, and MSE, MAE, medAE and SD values were lower and hence better than OMRON. It is now clearly observed that walking produced better results for the 20 cm and 60 cm range, which was previously inconclusive. Furthermore, a higher coefficient of determination (R-squared) proves that the radar can detect HR values which better fit the regression line. Here, R-squared value refers to the percentage of variation in the OMRON device’s HR readings that the radar can collectively explain as defined in (18)

(18) $$R^{2}=1-\frac{SS_{reg}}{SS_{tot}}=1-\frac{\sum_{i}{\left(y_{i}-\hat{y}\right)}^{2}}{\sum_{i}{\left(y_{i}-\bar{y}\right)}^{2}}$$

where $SS_{reg}$ is the residual sum of squared errors, $SS_{tot}$ is the total sum of squared errors, $y_{i}$ is a radar HR observation, $\hat{y}$ is the corresponding OMRON reading, and $\bar{y}$ is the mean of all radar observations.

#### 3.3.2. Validating Breathing Rate Values

Table 7 clearly concludes that BR values were best when a person was standing without making any movements for all distances measured in our experiment.

Hence, Table 6 and Table 7 show that HR values recorded during movements had better correlation with the reference device while BR values had large deviations from the mean. Since our radar measurement system only measures phase change within the same range bin, it is possible that bodily movements induced a change in the phase along the fast-time axis. Hence, phase changes due to breathing chest displacements (0.1–0.6 Hz) was easily discarded as noise.

### 3.4. Signal Processing and Arrhythmia Detection

#### 3.4.1. ECG Signal Processing

The MIT-BIH normal sinus dataset [33] consists of 18 single-lead ECG recordings, which includes five men between the age of 26 and 45 and women between 20 and 50. The MIT-BIH arrhythmia dataset [34] consists of two-lead ECG recordings of 47 subjects. Of the 47 readings, 15 readings were selected for training the ANN model. Both sets of signals were 10 s long, and each sample was subtracted by the baseline, whose result was divided by the gain. The signal specifications of the generated dataset are outlined in Table 8. Outputs of the signal processing steps as described in the previous section are plotted in Figure 6.

**Figure 8:**
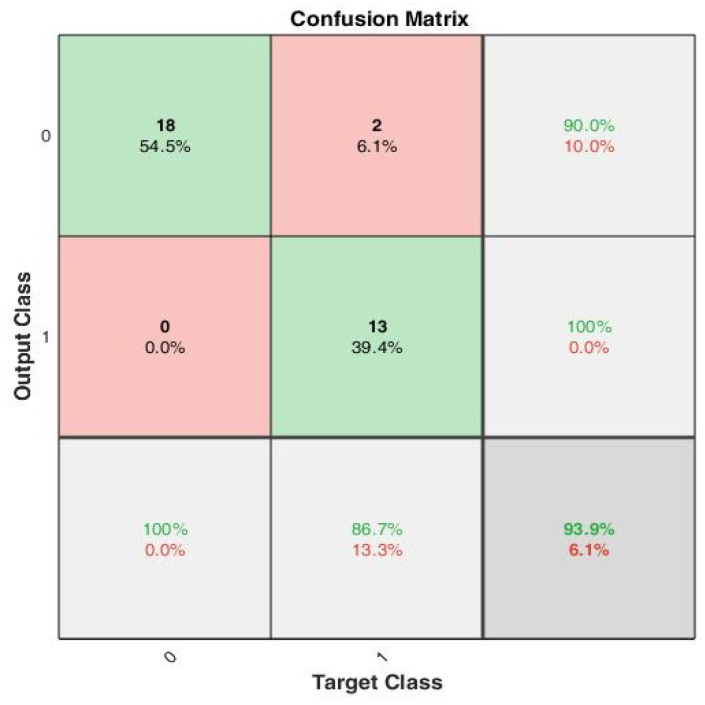
A confusion matrix that summarizes the model performance with true positivity rate and true negativity rate, with accuracy of 100%, 90% and 93.9%, respectively.

The peak-to-peak interval-based features were estimated using (13)–(17) to create the training set, which consists of 33 training examples. The data are split 70–30, i.e., 70% training, 15% validation and 15% test data for 10 epochs. The confusion plot in Figure 8 reveals that out of the 33 training examples, 18 were true negatives (TN), 2 false positives (FP), 13 true positives (TP) and 0 false negatives (FN).

**Figure 9:**
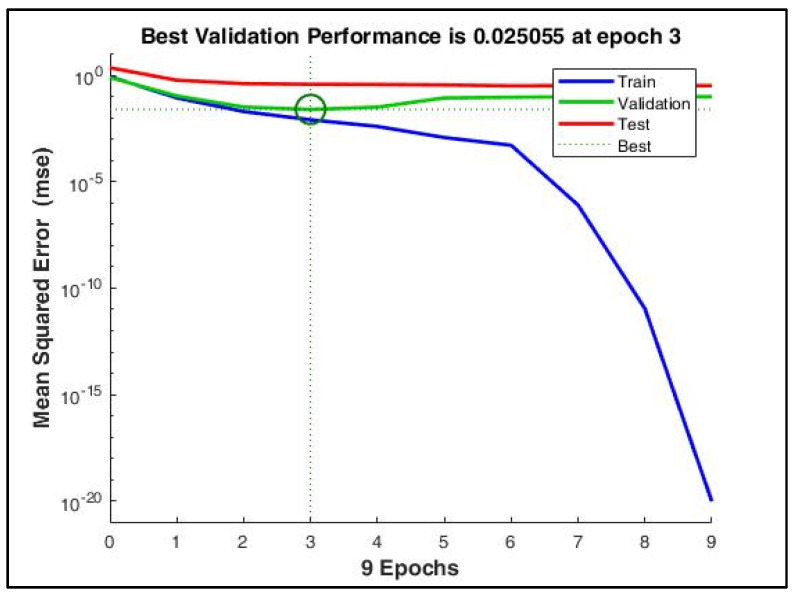
The mean squared error during training reduced to a very low value at the 9th epoch. However, with early stopping enabled, the best performance was obtained at the 3rd epoch when the validation MSE = 0.025.

**Figure 10:**
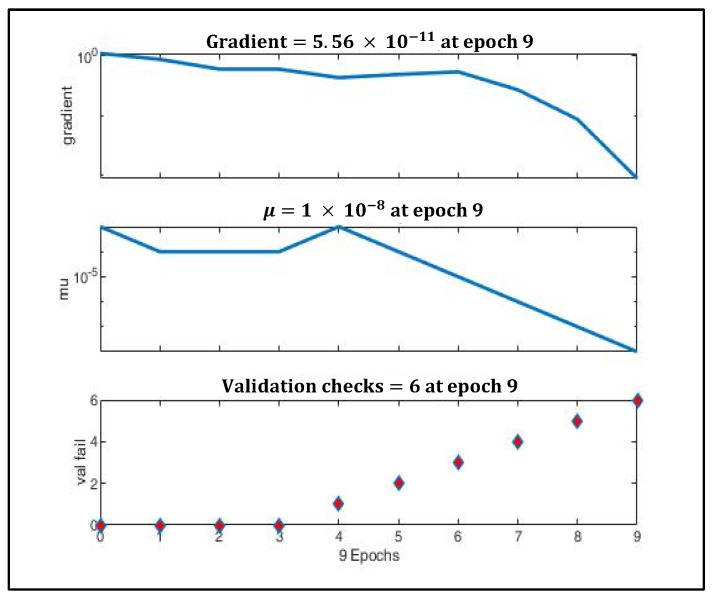
Gradient optimization using gradient descent and momentum.

Figure 9 depicts the gradual minimization of mean square error (MSE) during training. It was found that at the third epoch, MSE was minimized to 0.025 during validation. Figure 10 shows that the gradient value gradually decreased to nearly 0 at epoch nine, which signifies that the weights cannot be trained further.

The μ value (momentum) was reduced gradually to adaptively decrease the value of the gradient. To prevent the model from gradient overshooting, the momentum should reduce the rate at which the gradient value decreases when the MSE is approaching local minima.

#### 3.4.2. Radar Heartbeat Signal Processing

**Figure 11:**
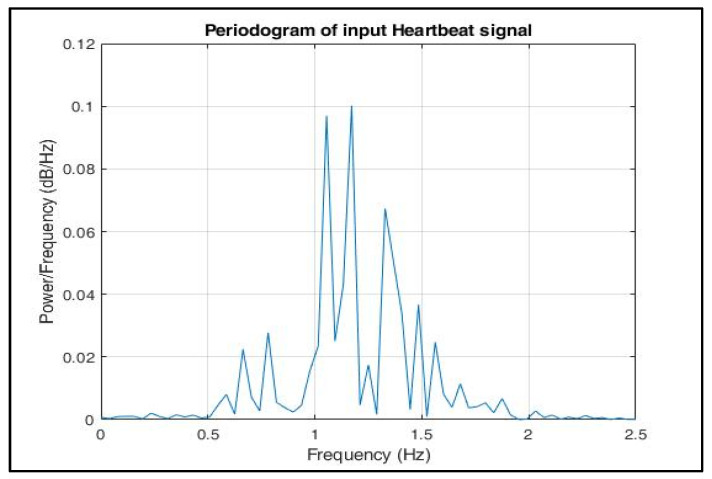
Periodogram of input heartbeat phase signal.

The periodogram in Figure 11 signifies that the signal consisted of components in the frequency range 0.6–2 Hz.

However, the exact frequency at which the maximum power was estimated was unclear. Since our training dataset was formed based on-RR interval-based features only and the R peak amplitudes, while modelling our radar signal (as described in Section 2), we can simply set each phase value equal to its mean. Hence, only the time duration of each peak phase value was extracted.

**Figure 12:**
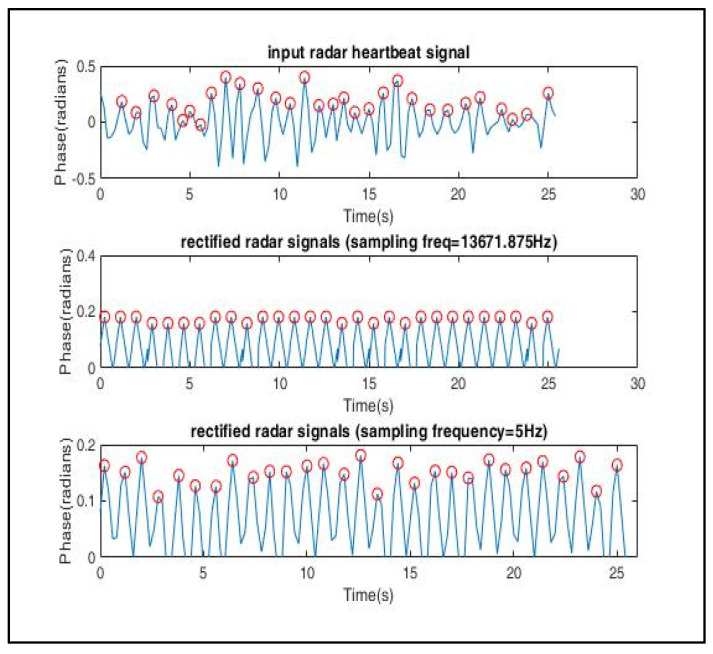
Signal reconstruction using symmetric triangular wave function, which was then down sampled to 5 Hz to match the sampling frequency of the ECG signals used in the training dataset.

**Figure 13:**
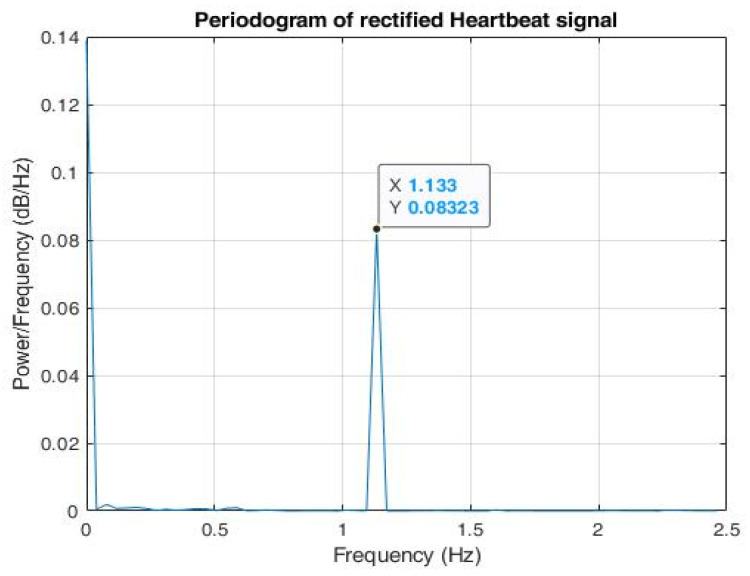
Periodogram of reconstructed phase signal shows that maximal power spectral density (PSD) lies in the frequency = 1.133 Hz.

A total of 100,000 data samples were utilized to model each triangular wave component. For the input radar signal as shown in Figure 12, n peaks were detected; hence, there were 100,000 × *n* data samples. Therefore, the sampling frequency was set as $Fs=\frac{100,000\times n}{25.6\;s}\;\mathrm{Hz}$. The signal was then down sampled to the desired sampling frequency, *Fs* = 5 Hz. The periodogram of the modelled signal as shown in Figure 13 clearly shows that the power spectral density (PSD) lay at frequency = 1.133 Hz, which lies within the expected frequency range of a heartbeat phase signal, i.e., 0.8–2 Hz. Hence, a well-constructed signal was generated whose peak values can be easily detected for testing the trained arrhythmia detection model.

**Figure 14:**
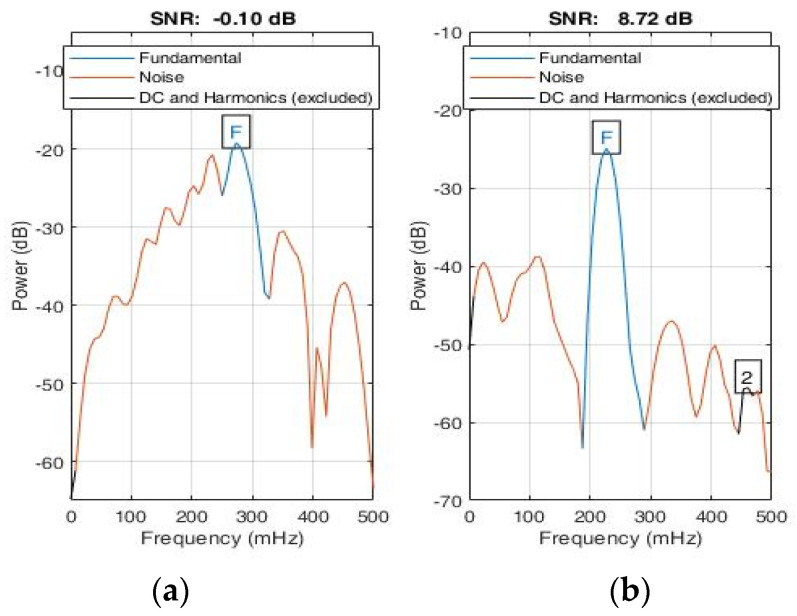
Signal-to-noise ratio (**a**) SNR of input signal, (**b**) SNR of reconstructed signal using symmetrical triangular QRS wave function.

In addition to the result obtained in Figure 14, a considerable improvement in the SNR value from −0.1 dB to 8.72 dB can be observed in Figure 13.

#### 3.4.3. Arrhythmia Detection Test

The trained neural network model was tested on unseen data generated by eight individuals. For every subject, 10 sets of observations were taken from the radar, and further peak-interval-based statistical features were extracted. The results are summarized in Table 9. However, it is important to note that our experiments were limited to testing only healthy individuals. The remaining seven subjects were tested on unseen ECG data from the MIT-BIH Arrhythmia Database.

## 4. Conclusions

An mm-wave FMCW radar-based non-contact vital signs monitoring system was implemented that continuously monitors the heart and respiratory rate. Based on the experiments carried out using the system, it was found that HR values estimated by the radar only deviated from our reference device by ±4. The effect of orientation and distance was statistically analysed (using number of outliers, SD, MSE and skewness) based on HR and BR values, and conclusive results show that right and left orientations were unsuitable for monitoring and the distance of measurement should be restricted to 120 cm. Furthermore, experiments on the effect of movement interestingly revealed that the HR recorded while walking had a better overall performance (in terms of MSE, SD, R-squared) than those recorded while the individual was stationary with respect to the reference device. However, standing in a stationary position is more favourable while recording breathing rates. To improve the overall reliability of arrhythmia detection, the heartbeat signal was rectified by representing them as triangular wave functions using Fourier series expansions. The resulting signals of the improved SNR value was then utilized to test an ECG-trained neural network model whose training features are statistical metrics based on RR-interval values. A 75% mean test accuracy was obtained for 15 observations that classify whether the heartbeat signals from a test subject were positively or negatively cardiac arrhythmic.

## Figures and Tables

**Table 1:** Vital signs measurement parameters.

Parameters	Value
Starting frequency	77 GHz
Slow axis sampling	20 Hz (chirps/s)
Chirp duration	50 µs
ADC sampling rate	2 Msps
Range resolution	4.3 cm
Transmitted power	10 dBm

**Table 2:** Model architecture where Kfold = 5, random seed = 42, loss function = MSE, optimizer = Levenberg–Marquardt algorithm, momentum = enabled, early stopping = 10 rounds, autotuning objective function = MSE, autotuning algorithm: grid search, BatchNorm = disabled.

Autotuned Parameters	Value
Input dense layer	8 nodes
Hidden dense layer	16 nodes
Output dense layer	1 node
Learning rate	0.01
L1 Regularization	0
L2 Regularization	0
Epochs	10

**Table 3:** Evaluation metrics to compare cuff-based and radar-based HR monitoring.

Metric	OMRON	Radar
Avg HR	74	74
Variance	13.95	8.5
STD	3.83	2.99
R^2^		0.164
Root mean square error (RMSE)		2.81
Mean absolute error (MAE)		1.9
Median absolute error (MedAE)		2

**Table 4:** Statistical evaluation of effect of orientation and distance on measurement.

Distance (cm)	Orientation	Vital Sign	Upper Bound	Lower Bound	Outliers	Mean	MSE	MAE	medAE	SD
30	front	HR	85.375	78.375	0	81.8	0.296	2.1	2	1.8
		BR	13.25	7.25	0	10.6	0.124	0.96	0.9	1.2
	right	HR	87.375	58.375	0	72.3	3.741	4.9	4	6.4
		BR	8	8	3	8.1	0.029	0.36	0.1	0.6
	left	HR	86.625	57.625	0	72.4	2.904	4.6	4	5.7
		BR	10.5	6.5	2	9.7	1.061	2.12	1.5	3.4
	back	HR	90.75	58.75	0	75.6	2.704	4.24	5	5.5
		BR	32.75	0.75	0	17.6	3.424	4.76	3.9	6.2
60	front	HR	92.125	67.125	0	80.1	3.129	4.46	4	5.9
		BR	14	6	0	10.4	0.144	1.12	1.4	1.3
	right	HR	103.25	39.25	0	72	9.6	8.8	8.5	10
		BR	15.875	4.875	1	10.7	0.701	2.24	1.7	2.8
	left	HR	91	63	1	76	4.42	5.2	4	7
		BR	19.625	2.625	1	11.9	2.009	3.66	2.9	4.7
	back	HR	81.75	67.75	3	74.1	4.269	4.7	2	6.9
		BR	29.5	3.5	0	16.5	1.905	3.9	3.5	4.6
90	front	HR	89.5	63.5	0	75.5	3.285	4.6	4	6
		BR	10.5	6.5	1	8.8	0.076	0.64	0.5	0.9
	right	HR	91.375	54.375	1	72.2	6.956	6.6	4.7	8.8
		BR	16.5	4.5	2	12.9	4.649	5.24	3.9	7.2
	left	HR	94.375	63.375	0	78.6	3.004	4.92	3.6	5.8
		BR	24.75	−3.25	1	12.1	7.029	6.54	4.6	8.8
	back	HR	86.125	61.125	0	73.7	1.721	3.5	3.3	4.4
		BR	30.375	−4.625	0	13.2	1.996	3.76	4.8	4.7
120	front	HR	104.25	56.25	0	78.6	8.624	7.72	5.1	9.8
		BR	14	6	0	9.9	0.129	1.1	1.1	1.2
	right	HR	86.375	55.375	0	71.5	2.705	4.4	3.5	5.5
		BR	19.5	1.5	0	11	1.5	3.4	3	4.1
	left	HR	93.5	57.5	1	73.7	9.841	7.42	5.5	10
		BR	23.25	−0.75	2	12.9	5.169	5.86	4.4	7.6
	back	HR	87.875	52.875	0	69	4.56	5.4	5.5	7.1
		BR	36.25	−5.75	0	15.2	2.636	4.6	5.8	5.4
150	front	HR	88.875	63.875	0	75.9	1.949	3.88	3.5	4.7
		BR	12.75	6.75	0	9.6	0.144	1.04	0.6	1.3
	right	HR	87.125	54.125	1	70.8	8.296	7.4	4.5	9.6
		BR	19.875	−9.125	0	7.3	3.041	4.44	4.5	5.8
	left	HR	92.375	61.375	1	74.8	6.056	5.96	5	8.2
		BR	31.25	−18.75	0	7.9	6.509	6.5	7	8.5
	back	HR	91.25	57.25	0	73.7	2.721	4.7	4.5	5.5
		BR	34.625	−2.375	0	16.8	5.156	6.16	5	7.6

**Table 5:** Statistical evaluation of effect of movement.

Distance (cm)	Activity	Vital Sign	Upper Bound	Lower Bound	Outliers	Mean	MSE	MAE	MedAE	SD
20	standing	HR	74.88	71.88	4	72	62.8	5.4	1.5	8.35
		BR	10.5	6.5	1	9	1.04	0.8	0.6	1.07
	walking	HR	85.5	59.5	0	73	14.6	3.48	3.5	4.03
		BR	28.88	3.875	2	18	52.3	5.68	4.4	7.62
40	standing	HR	77.5	65.5	0	72	6.56	2.2	1.8	2.7
		BR	16.13	3.125	1	11	18.6	3.32	2.8	4.54
	walking	HR	74.5	62.5	2	70	23.7	3.36	2.1	5.13
		BR	34.5	−3.5	0	17	49.6	5.4	4.5	7.42
60	standing	HR	88	64	2	74	38.4	4.84	4.2	6.53
		BR	16.5	4.5	1	11	13.8	2.56	2	3.92
	walking	HR	81.88	68.88	0	75	10.5	2.66	1.9	3.41
		BR	29.63	0.625	0	14	23	3.88	4.5	5.06

**Table 6:** Performance analysis of heart rate with respect to Omron device.

Distance	Activity	Vital Sign	Mean	MSE	MAE	medAE	SD	R Square
20	standing	HR	72	97.61	7.72	4.9	8.353	0.285137
	walking	HR	73	42.73	5.3	3.9	4.033	0.6193151
40	standing	HR	72	43.77	6.1	5.9	2.7	0.833473
	walking	HR	70	84.53	8.22	7.9	5.131	0.688605
60	standing	HR	74	55.17	5.36	2.5	6.529	0.2274384
	walking	HR	75	18.33	3.22	1.9	3.414	0.3641268

**Table 7:** Performance analysis of breathing rate (with and without moving).

Distance	Activity	Vital Sign	Mean	MSE	MAE	medAE	SD
20	standing	BR	9	1.04	0.8	0.6	1.07497
	walking	BR	18	52.29	5.68	4.4	7.62234
40	standing	BR	11	18.56	3.32	2.8	4.54117
	walking	BR	17	49.6	5.4	4.5	7.42369
60	standing	BR	11	13.81	2.56	2	3.9172
	walking	BR	14	23.04	3.88	4.5	5.05964

**Table 8:** ECG signal specifications of the training dataset.

Specifications	Normal Sinus Dataset	Arrhythmia Dataset
Sampling frequency (Hz)	128	360
Number of samples	1280	3600
Gain (adu/mV)	200	200
Baseline	0	1024

**Table 9:** Testing accuracies of 15 subjects: the average test accuracy was estimated to be 75% for 15 subjects. The coefficient of determination of 0.876 for the trained dataset is justified.

Subject ID	Age	Gender	Testing Accuracy	Predicted	Actual	False Positives
1	22	male	60%	Normal	Normal	4
2	24	male	90%	Normal	Normal	1
3	23	male	60%	Normal	Normal	4
4	30	female	80%	Normal	Normal	2
5	20	male	100%	Normal	Normal	0
6	25	male	60%	Normal	Normal	4
7	50	female	90%	Normal	Normal	1
8	45	female	100%	Normal	Normal	0
9	68	male	60%	Arrhythmia	Arrhythmia	4
10	69	male	80%	Arrhythmia	Arrhythmia	2
11	69	male	70%	Arrhythmia	Arrhythmia	3
12	51	female	20%	Arrhythmia	Arrhythmia	8
13	83	female	80%	Arrhythmia	Arrhythmia	2
14	51	male	80%	Arrhythmia	Arrhythmia	2
15	63	female	90%	Arrhythmia	Arrhythmia	1

## Data Availability

The normal sinus and arrhythmia datasets were taken from the open-source MIT-BIH repository from https://archive.physionet.org/physiobank/database/nsrdb/ (accessed on 1 November 2019) and https://physionet.org/content/mitdb/1.0.0/ (accessed on 1 November 2019), respectively.
